# UniCon: A unified star-operation to efficiently find connected components on a cluster of commodity hardware

**DOI:** 10.1371/journal.pone.0277527

**Published:** 2022-11-30

**Authors:** Chaeeun Kim, Changhun Han, Ha-Myung Park

**Affiliations:** Kookmin University, Seoul, Republic of Korea; Hankuk University of Foreign Studies, KOREA, REPUBLIC OF

## Abstract

With a cluster of commodity hardware, how can we efficiently find all connected components of an enormous graph containing hundreds of billions of nodes and edges? The problem of finding connected components has been used in various applications such as pattern recognition, reachability indexing, graph compression, graph partitioning, and random walk. Several studies have been proposed to efficiently find connected components in various environments. Most existing single-machine and distributed-memory algorithms are limited in scalability as they have to load all data generated during the process into the main memory; they require expensive machines with vast memory capacities to handle large graphs. Several MapReduce algorithms try to handle large graphs by exploiting distributed storage but fail due to data explosion problems, which is a phenomenon that significantly increases the size of data as the computation proceeds. The latest MapReduce algorithms resolve the problem by proposing two distinguishing star-operations and executing them alternately, while the star-operations still cause massive network traffic as a star-operation is a distributed operation that connects each node to its smallest neighbor. In this paper, we unite the two star-operations into a single operation, namely UniStar, and propose UniCon, a new distributed algorithm for finding connected components in enormous graphs using UniStar. The partition-aware processing of UniStar effectively resolves the data explosion problems. We further optimize UniStar by filtering dispensable edges and exploiting a hybrid data structure. Experimental results with a cluster of 10 cheap machines each of which is equipped with Intel Xeon E3-1220 CPU (4-cores at 3.10GHz), 16GB RAM, and 2 SSDs of 1TB show that UniCon is up to 13 times faster than competitors on real-world graphs. UniCon succeeds in processing a tremendous graph with 129 billion edges, which is up to 4096 times larger than graphs competitors can process.

## Introduction

Given a large graph containing hundreds of billions of nodes and edges, how can we find all connected components efficiently on a cluster of commodity hardware? A connected component in a graph is a maximal subset of nodes connected by paths. Finding connected components is one of the most important tasks in the field of graph analysis with various applications including pattern recognition [1, 2], reachability indexing [3–5], graph compression [6–8], graph partitioning [9–11], random walk [12], etc. Meanwhile, billion to trillion-scale graphs have emerged recently, which are very challenging to handle because of the enormity.

Various methods have been proposed to efficiently find connected components in large graphs, which are common these days with the expansion of data on the Web. Parallel algorithms [13–15], external algorithms [16–19], and distributed-memory algorithms [20–25] run quickly on moderate-sized graphs. However, these algorithms fail when the graph is large because they have to load all data generated during the process, including the entire input graph, into the main memory (see Fig 14). Like in [15, 26] and [25], some algorithms reportedly handle a hundred billion scale graphs by exploiting expensive machines, but such machines are unaffordable for common data scientists.

Several MapReduce algorithms [26–31] try to handle large graphs by exploiting distributed storage but fail because they execute distributed operations a lot or suffer from data explosion problems, which significantly increase the size of data as the computation proceeds which leads to a massive disk and network I/O. To resolve these problems, recent MapReduce algorithms [32–34] propose two distinguishing star-operations and conduct them alternately, where a star-operation is a distributed operation that transforms the input graph into another one keeping the connectivity. Then, our question is: *Is there any way to improve the performance of the MapReduce algorithms by merging the two star-operations into one?* The data explosion problem occurs again if we combine two star-operations carelessly.

In this paper, we propose UniStar, a unified star-operation, and UniCon, a new distributed algorithm using UniStar. UniStar avoids the data explosion problem by *partition-aware processing*, which partitions nodes and processes nodes in each partition together. We further optimize UniStar in two ways: 1) *filtering dispensable edges* to reduce intermediate data and 2) minimizing the memory consumption in workers by a custom data structure *HybridMap*. We summarize the main contributions of this paper as follows:

- **Algorithm.** We propose UniStar, a unified star-operation avoiding data explosion problem. We also propose UniCon, a fast and scalable distributed algorithm using UniStar for finding connected components in an enormous graph.
- **Theory.** We prove the correctness and various properties of UniCon. We guarantee that the expected memory usage of a worker by UniCon is *O*((|*V*| + |*E*|)/*ρ*) where |*V*|, |*E*|, and *ρ* are the numbers of nodes, edges, and partitions, respectively.
- **Experiment.** Extensive experiments show that UniCon outperforms the state-of-the-art distributed algorithms; UniCon runs as fast as distributed-memory algorithms and succeeds in processing a tremendous graph with 129 billion edges, using only 10 cheap machines each of which is equipped with Intel Xeon E3-1220 CPU (4-cores at 3.10GHz), 16GB RAM, and 2 SSDs of 1TB.

The codes and datasets used in this paper are available in https://github.com/UniCon2021/UniCon.

## Related work

In this section, we review and compare existing methods for finding connected components in three categories: single-machine algorithms, distributed-memory algorithms, and MapReduce algorithms. We also describe how the proposed method is improved over the existing algorithms.

### Single-machine algorithms

Traditional graph traversal algorithms such as breadth-first search and depth-first search find connected components in linear time on the size of the graph. Loading the entire graph into the memory, they require *O*(|*V*| + |*E*|) memory space where |*V*| and |*E*| are the numbers of nodes and edges in the graph, respectively. Union-Find based algorithms [35–37] reduce required memory space to *O*(|*V*|) by exploiting a parent pointer tree data structure. Our proposed method UniCon uses Rem [37], a Union-Find based single machine algorithm, with modification as a module. Multi-core algorithms [13–15] reduce the running time by exploiting multi-core CPUs. ConnectIt [15] is the state-of-the-art multi-core algorithm showing the fastest performance by advanced optimization techniques such as edge sampling, tree linking, and tree compression. However, the multi-core algorithms, including ConnectIt, are limited in scalability because they require loading the entire graph into the main memory. To process a large graph using the above multi-core algorithms, we have to prepare an expensive machine with a huge memory capacity. For example, in [15], the authors use a machine with 72 cores and 1TB memory to process billion-scale graphs; our experiments show that ConnectIt fails to process large graphs on a commodity machine (see Section “Results on Real-world Datasets”).

### Distributed-memory algorithms

For the purpose of improving the speed and scalability, distributed-memory algorithms exploit the main memory of multiple machines to store all the input and the intermediate data generated during the process. Pregel-like systems [18, 20–23, 38, 39] describe graph algorithms, including connected component computation, as a set of node-centric operations that propagate the value of a node to neighboring nodes repeatedly. FastSV [25], the state-of-the-art distributed-memory algorithm, and LACC [24] compute connected components in a linear algebraic way; they implement the Awerbuch-Shiloach algorithm [40] using the Combinatorial BLAS library [41], which provides several primitives to represent graph algorithms. However, the above distributed-memory algorithms fail when the intermediate data does not fit into the memory. To process a large graph with distributed-memory algorithms, we need an expensive cluster with massive memory capacity. For example, LACC and FastSV use Cray XC40, the supercomputer composed of more than 4,000 nodes (262,000 cores, 360 TB main memory), to process a graph of 50 billion edges. We show that LACC and FastSV fail to process large graphs on a cluster of commodity hardware (see Section “Results on Real-world Datasets”).

### MapReduce algorithms

MapReduce [42] is a framework for processing large data using a cluster that consists of multiple commodity machines. While distributed-memory algorithms are limited to moderate-sized graphs, MapReduce is suitable for handling enormous graphs as it processes data in an I/O efficient manner on a distributed file system. Several MapReduce algorithms [26–30, 32–34] have been proposed to find connected components in enormous graphs. Pegasus [27] propagates the label of each node to its neighbors using a distributed operation each round. The number of rounds required by Pegasus is *O*(*d*) where *d* is the diameter of the graph. As each distributed operation takes non-trivial time, Pegasus does not scale well to large graphs. Hash-Greater-to-Min [28] reduces the number of rounds to *O*(*log*|*V*|), while Hash-to-Min, proposed in the same paper, runs faster in practice. Hash-to-min builds initial clusters each of which consists of a node and its neighbors, and then unions the clusters each round. Hash-to-Min, however, suffers from the problems of data explosion and load balancing. The alternating algorithm [32] resolves the data explosion problem by dividing the union operation of Hash-to-Min into two distributed operations, namely star-operation, and by executing them alternately. PACC [34] resolves the load balancing problem by redesigning the star-operations to partition the nodes; however, PACC still generates a large amount of intermediate data as it alternates the two star-operations as the alternating algorithm does. Note that our method UniCon elaborately unifies the two distributed operations to reduce the amount of intermediate data without load balancing problems. Cracker [31] also improves on the alternating algorithm via vertex pruning and edge reduction. Stergiou et al. [26] propose a label-propagation based distributed method guarantees a logarithmic round number by shortcutting. The algorithm is reported to handle the largest dataset to date exploiting a large-scale cluster that contains 5000 workers, each of which has 128GB memory. Unfortunately, the algorithm is not tested in our experiments because it is not publicly available and not reproducible; it is implemented on Yahoo’s private graph processing system. We believe that the method can’t handle large graphs on commodity machines because it requires loading the entire graph on memory every round to examine every edge in the graph at each iteration as described in [26].

## Preliminaries

In this section, we define the problem of finding all connected components. Symbols frequently used in this paper are listed in Table 1.

**Table 1:** Table of symbols.

Symbol	Definition
*G* = (*V*, *E*)	Undirected graph with node set *V* and edge set *E*
*u*, *v*, *w*	Nodes
(*u*, *v*)	Edge between *u* and *v* such that *u* > *v*
Λ(*u*, *G*)	Connected component containing node *u* in *G*
*m*(*S*)	Most preceding node in node set *S*
Γ^−^(*u*, *G*)	Set of preceding neighbors of *u* in graph *G*: {*v*|(*u*, *v*)∈*E*}
Γ^+^(*u*, *G*)	Set of following neighbors of *u* in graph *G*: {*v*|(*v*, *u*)∈*E*}
Γ(*u*, *G*)	Set of all neighbors of *u* in graph *G*: Γ^+^(*u*, *G*)∪Γ^−^(*u*, *G*)
Γ^⋆^(*u*, *G*)	Set of neighbors *v* of node *u* in graph *G* = (*V*, *E*) such that the edge between *u* and *v* is not intact
*ρ*	Number of partitions
[*ρ*]	Set of partition ids: {0, …, *ρ* − 1}
*ξ*(*u*)	Partition of node *u*
[*S*]_*i*_	*i*-th partition of node set *S*: {*v* ∈ *S*|*ξ*(*v*) = *i*}

### Problem definition

Let *G* = (*V*, *E*) denote an undirected graph where *V* and *E* are the sets of nodes and edges, respectively. The nodes in *V* are totally ordered; *u* < *v* indicates that *u* precedes *v* (or *v* follows *u*). An edge between two nodes *u* and *v* is denoted as an ordered pair: (*u*, *v*) if *u* > *v*, (*v*, *u*) otherwise. We say two nodes *u* and *v* are *connected* if *G* contains a path from *u* to *v*. A connected component, shortly a component, of *G* is a maximal subset of *V* where all pairs are connected in *G*. Every node belongs to exactly one component. We denote the component containing node *u* by Λ(*u*, *G*). The problem of interest in this paper is finding all components in a given graph. This problem is equivalent to mapping each node *u* to the representative node in Λ(*u*, *G*). Even though any node in a component can be the representative node, we consider the most preceding node in the component to be the representative node. For a node set *S*, we denote the most preceding node in *S* by *m*(*S*). Then, we formally define the problem of finding connected components as follows:

**Definition 1**. *(Finding All Connected Components) Given an undirected graph G* = (*V*, *E*), *the problem of finding all connected components is to map each node u* ∈ *V to the most preceding node m*(Λ(*u*, *G*)) *in the component containing u*.

We define several symbols that are used to describe our method. For a graph *G* = (*V*, *E*) and a node *u* ∈ *V*, we denote by Γ^−^(*u*, *G*) = {*v*|(*u*, *v*)∈*E*} the set of preceding neighbors of *u*. Similarly, we denote by Γ^+^(*u*, *G*) = {*v*|(*v*, *u*)∈*E*} the set of following neighbors of *u*. Γ(*u*, *G*) = Γ^−^(*u*, *G*)∪Γ^+^(*u*, *G*) denotes the set of all neighbors of *u*. UniCon partitions the nodes by a random hash function *ξ*: *V* → [*ρ*] where *ρ* is the number of partitions and [*ρ*] = {0, …, *ρ* − 1} is the set of partition ids. *ξ*(*u*) is the partition id of node *u*. For a node set *S*, [*S*]_*i*_ = {*v* ∈ *S*|*ξ*(*v*) = *i*} denotes the *i*-th partition of node set *S*. Each partition [*V*]_*i*_ of node set *V* is totally ordered.

## Proposed method

In this section, we propose UniCon, a new distributed algorithm for finding connected components. UniCon achieves high-speed and high-scalability by dealing with the following challenges.

1. To avoid data explosion problems, existing MapReduce algorithms [32–34] divide the union operation of Hash-to-Min [28] into two star-operations, which still transfer massive data via the network. **How do we reunite two star-operations into one while resolving data explosion problems?** We propose a new star-operation UniStar that alleviates data explosion problems by **partition-aware processing**; it removes duplicate edges in each partition and leads to early convergence as nodes jump to near the representative node through the edges in each partition. (Section “UniStar: The Unified Star Operation”)
2. UniStar reads and writes all edges of the input graph but most edges do not change anymore after several rounds. **How do we figure out such edges during the process and filter out them to minimize the size of data I/O?** We elaborately design three types of edges that no longer contribute to updating the graph. UniCon **filters out such dispensable edges** and reduces the intermediate data size significantly. (Section “UniStar-opt: Filtering Out Dispensable Edge”)
3. In UniStar, each worker uses a data structure to keep the preceding node for each node. It is easy to run out of memory if the data structure is inadequately designed, especially on commodity machines. **How do we efficiently design the data structure and guarantee the memory consumption of UniCon?** **A hybrid data structure** of an array and a hash table ensures that the expected memory size required by each worker is *O*((|*V*| + |*E*|)/*ρ*) while showing the fast performance in practice, where *ρ* is the number of partitions. (Section “A Hybrid Map Data Structure”)

**Fig 1:**
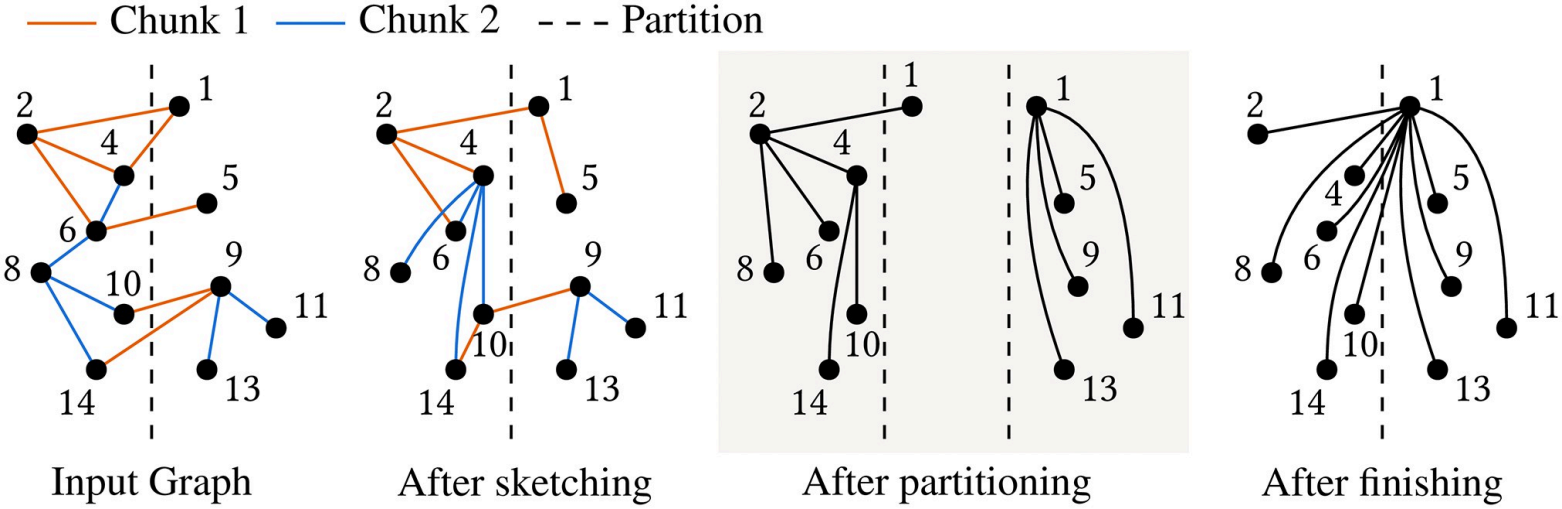
An example showing the input and output for each step of UniCon.

We describe the overall structure of UniCon. Algorithm 1 is the pseudocode of UniCon. UniCon consists of three steps: sketching, partitioning, and finishing. Fig 1 is an example showing the input and output of each step. The sketching step, proposed in [34], computes connected components on each chunk of the input graph to reduce the graph size and does a load balancing work (line 2), where a chunk is a subset of edges existing consecutively in storage. In the partitioning step, UniCon partitions the input graph into the number of partitions *ρ* overlapping subgraphs by iteratively running the unified star-operation UniStar or the optimized version UniStar-opt (lines 3-9). If the number of input edges is less than a threshold *τ*, UniCon runs Rem instead of UniStar to reduce the number of rounds (lines 4-8). After the partitioning step, the nodes in each subgraph are connected to the representative node by paths so that the finishing step computes connected components correctly by independently processing each subgraph using Rem (line 10). We describe UniStar in Section “UniStar: The Unified Star Operation” and UniStar-opt in Section “UniStar-opt: Filtering Out Dispensable Edges” in detail.

**Algorithm 1:** UniCon

**Input:** *E* as an edge list and *V* where *G* = (*V*, *E*)

**Output:** {(*u*, *m*(Λ(*u*, *G*)))|*u* ∈ *V*}

**1** *F* ← ∅;

**2** *E* ← Sketching(*E*)          // of [34]

**3 repeat**

**4**  **if** |*E*| > *τ* **then**

**5**   *E*, ‘sep’ ← UniStar-opt(*E*)     // Algorithm 2

**6**   *F* ← *F*∪ ‘sep’;

**7**  **else**

**8**   *E* ← Rem(*E*)          // of [37]

**9** **until** *Convergence*;

**10** **return** Finishing(*V*, *E*, *F*)

### UniStar: The unified star operation

We first demonstrate that combining two star-operations in a simple way causes data explosion problems. After that, we propose *UniStar*, a unified star-operation that resolves the data explosion problems.

#### UniStar-naïve

**Fig 2:**
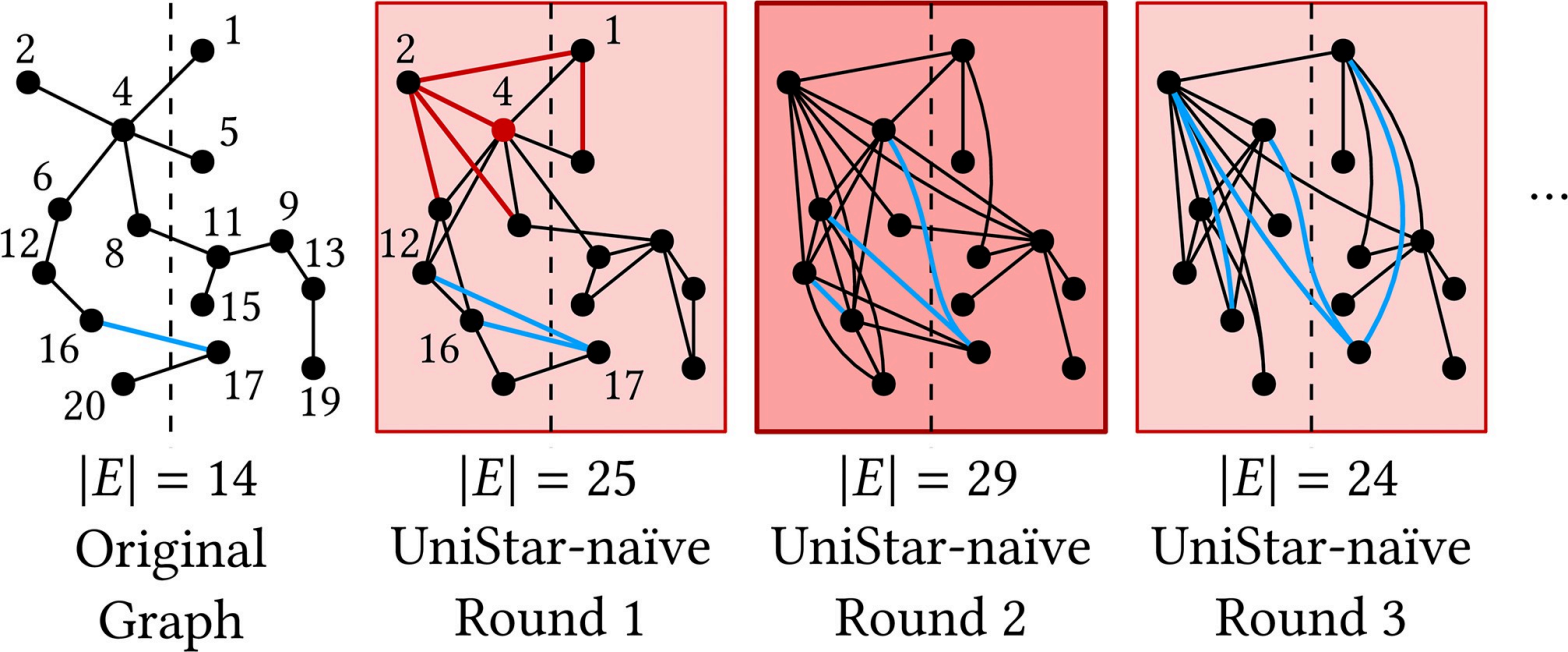
UniStar-naïve causes a data explosion problem. The more edges, the darker the background color in red. The blue lines show how edge (17, 16) propagates; the edge is copied every round. The dashed-line is the partition.

One simple method to combine two distributed operations is UniStar-naïve. For each node *u*, UniStar-naïve connects each neighbor *v* ∈ Γ(*u*, *G*) ∪ {*u*} to *m*(Γ(*u*, *G*) ∪ {*u*}), but if *v* ≠ *m*([Γ(*u*, *G*) ∪ {*u*}]_*ξ*(*v*)_) then connects *v* to *m*([Γ(*u*, *G*) ∪ {*u*}]_*ξ*(*v*)_) for load balancing, like in [33]. In round 1 of Fig 2, for example, the neighbors Γ(4, *G*) ∪ {4} = {1, 2, 4, 5, 6, 8} of node 4 are connected to *m*([Γ(4, *G*) ∪ {4}]_0_) = 2 or *m*([Γ(4, *G*) ∪ {4}]_1_) = 1, and *m*([Γ(4, *G*) ∪ {4}]_0_) = 2 is connected to *m*(Γ(4, *G*) ∪ {4}) = 1.

Simply combining two distributed operations, however, causes a data explosion problem, which prolongs the running time significantly or leads to failure. Fig 2 shows the demonstration of the data explosion problem caused by UniStar-naïve. The number of edges rises to 29 in Round 2, while the number of input edges is 14. The reason the number of edges increases is that each edge is processed on both side nodes. For example, UniStar-naïve of round 1 copies edge (17, 16) in the original graph to edges (17, 12) and (17, 16) since *m*(Γ(16, *G*) ∪ {16}) = 12 and *m*(Γ(17, *G*) ∪ {17}) = 16. The copied edges are copied again and again in subsequent rounds, causing the data explosion problem.

#### UniStar

UniStar avoids the data explosion problem in two ways: *partition-aware processing* and *excluding intact-edge*.

**Fig 3:**
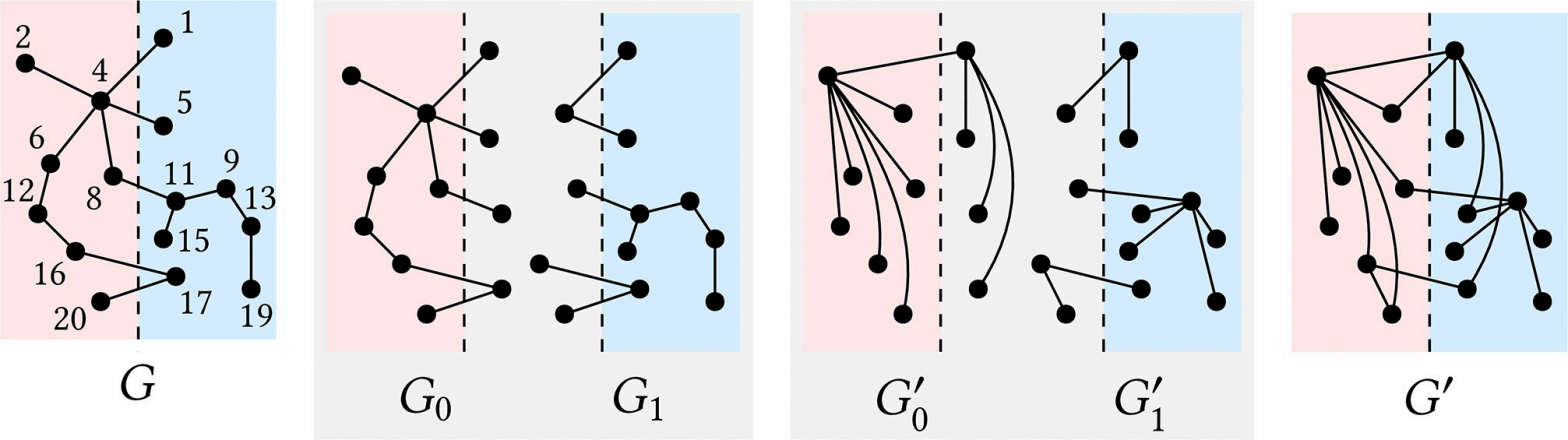
An example of UniStar when the number of partitions *ρ* = 2. Red and blue areas represent 0 and 1, respectively. UniStar divides *G* into overlapping subgraphs *G*_0_ and *G*_1_, computes $G_{0}^{\prime }$ from *G*_0_ by connecting each node *u* ∈ *V*_0_ to *m*(Λ(*u*, *G*_0_)) = 1 or *m*([Λ(*u*, *G*_0_)]_*ξ*(*u*)_) = 2, and computes $G_{1}^{\prime }$ in the same way. Then, *G*′ is the union of $G_{0}^{\prime }$ and $G_{1}^{\prime }$.

**Partition-aware processing.** For each partition *i*, *partition-aware processing* is to handle the nodes in partition *i* and their incident edges together on the same machine. Partition-aware processing has two advantages; it significantly reduces the number of edges by removing duplicate edges made in each partition and accelerates convergence by providing opportunities for each node *u* to jump to near *m*(Λ(*u*, *G*)) through the edges in each partition. Let *G*_*i*_ = (*V*_*i*_, *E*_*i*_) be the subgraph of *G* induced by the set *E*_*i*_ of edges incident to the nodes in partition *i*. UniStar computes $G_{i}^{\prime }$ by connecting each node *u* to *m*(Λ(*u*, *G*_*i*_)), but if *u* ≠ *m*([Λ(*u*, *G*_*i*_)]_*ξ*(*u*)_) then *u* to *m*([Λ(*u*, *G*_*i*_)]_*ξ*(*u*)_) for load balancing where Λ(*u*, *G*_*i*_) is *G*_*i*_’s connected component that contains *u*. Then, the output graph of UniStar is *G*′ = (*V*′, *E*′) where $V^{\prime }={⋃}_{i\in [\rho ]}V_{i}^{\prime }$ and $E^{\prime }={⋃}_{i\in [\rho ]}E_{i}^{\prime }$. Fig 3 shows an illustration of UniStar when the number *ρ* of partitions is 2. In this example, nodes 20 and 2 in $G_{0}^{\prime }$ are connected to nodes 2 and 1, respectively, because Λ(20, *G*_0_) = {1, 2, 4, 5, 6, 8, 11, 12, 16, 17, 20}, *m*([Λ(20, *G*_0_)]_*ξ*(20)_) = 2, and *m*(Λ(2, *G*_0_)) = 1.

**Theorem 1**. *The output graph G*′ = (*V*′, *E*′) *of UniStar has the same connectivity as the input graph G* = (*V*, *E*).

*Proof*. If nodes *u* and *v* in *G*_*i*_ are connected by a path, then *m*(Λ(*u*, *G*_*i*_)) = *m*(Λ(*v*, *G*_*i*_)). All nodes *u* in $G_{i}^{\prime }$ are connected to *m*(Λ(*u*, *G*_*i*_)) directly or through *m*([Λ(*u*, *G*_*i*_)]_*ξ*(*u*)_). It indicates that *G*_*i*_ and $G_{i}^{\prime }$ have the same connectivity. *G* and *G*′ also have the same connectivity as *E* = ⋃_*i*∈[*ρ*]_ *E*_*i*_ and $E^{\prime }={⋃}_{i\in [\rho ]}E_{i}^{\prime }$ by definition.

**Fig 4:**
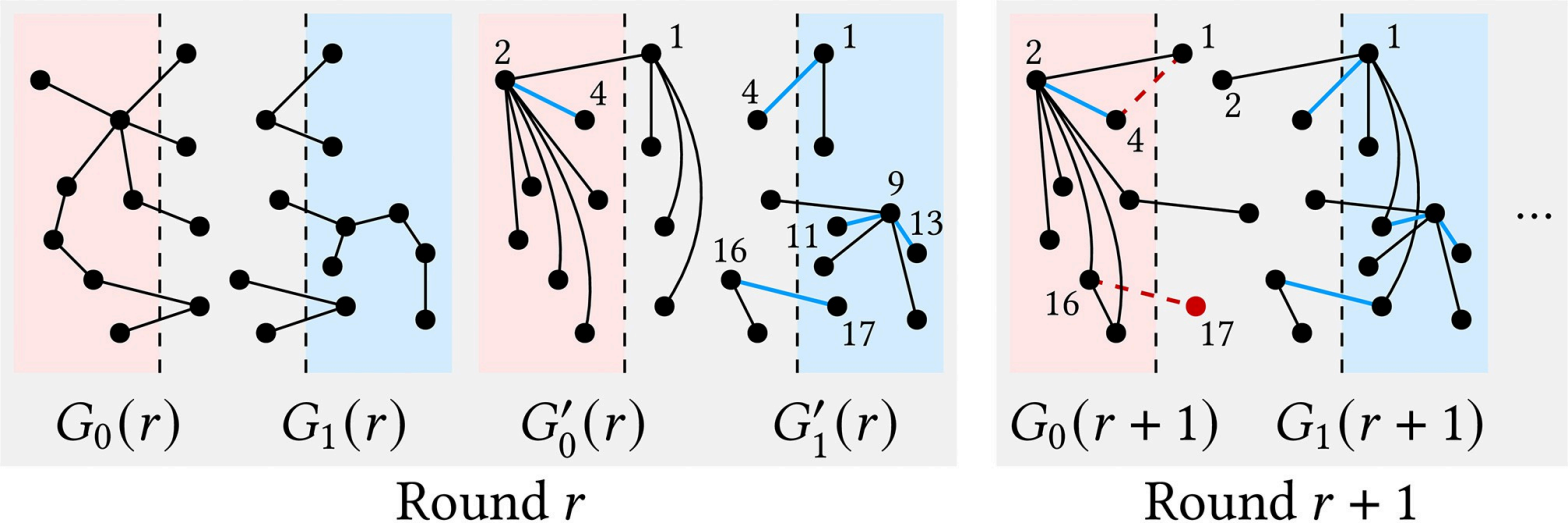
Intact edges in round *r* are marked in blue. The output of UniStar in round *r* becomes the input in round *r* + 1. Intact edge (*u*, *v*) in round *r* such that *ξ*(*u*)≠*ξ*(*v*) is excluded in a subgraph of round *r* + 1. The excluded intact edges are marked with red dashed lines.

**Excluding intact-edge.** UniStar reduces the amount of data to process by excluding several *intact edges* when dividing the input graph *G* into overlapping subgraphs *G*_*i*_ for *i* ∈ [*ρ*]. We say an edge is *intact* if the edge has not changed when transforming *G*_*i*_ to $G_{i}^{\prime }$, and we let $I_{i}=E_{i}^{\prime }\cap E_{i}$ be the set of intact edges from partition *i*. An intact edge (*u*, *v*)∈*I*_*ξ*(*v*)_ for *ξ*(*u*)≠*ξ*(*v*) implies that *u* has no path to another node through node *v*, and thus UniStar excludes the edge from *G*_*ξ*(*u*)_ in the next round. In Fig 4, for example, blue edges in round *r* are intact, and the intact edge sets are *I*_0_ = {(4, 2)} and *I*_1_ = {(4, 1), (11, 9), (13, 9), (17, 16)}. Let *G*(*r*), *G*′(*r*), *G*_*i*_(*r*), and $G_{i}^{\prime }\left(r\right)$ be *G*, *G*′, *G*_*i*_, and $G_{i}^{\prime }$ in round *r*, respectively. Similarly, Let *I*_*i*_(*r*) be *I*_*i*_ in round *r*. The output of round *r* is the input of round *r* + 1, i.e., *G*′(*r*) = *G*(*r*+ 1). UniStar divides *G*(*r*+ 1) into *G*_*i*_(*r*+ 1) for *i* ∈ [*ρ*], and each edge (*u*, *v*) in *G*(*r* + 1) exists in two subgraphs *G*_*ξ*(*u*)_(*r*+ 1) and *G*_*ξ*(*v*)_(*r*+ 1) if *ξ*(*u*)≠*ξ*(*v*). If edge (*u*, *v*) is an intact edge in *I*_*i*_(*r*), meanwhile, UniStar excludes (*u*, *v*) from *G*_*j*_(*r*+ 1) for *j* ≠ *i*. In Fig 4, edge $\left(2,1\right)\in G_{0}^{\prime }\left(r\right)\backslash I_{0}\left(r\right)$ exists in both *G*_0_(*r* + 1) and *G*_1_(*r* + 1), but edge (4, 1)∈*I*_1_(*r*) does only in *G*_1_(*r* + 1) and is excluded from *G*_0_(*r* + 1).

A MapReduce version of UniStar is listed in Algorithm 2 (ignore the blue lines). Given an edge (*u*, *v*) such that *u* > *v*, the map function of UniStar emits 〈*ξ*(*u*);(*u*, *v*)〉 and 〈*ξ*(*v*);(*u*, *v*)〉 (lines 1-4) so that the input of the reduce function is the edge set *E*_*i*_. If node *u* (or *v*) has a tag that tells the edge (*u*, *v*) is intact in the previous round, UniStar doesn’t emit 〈*ξ*(*u*);(*u*, *v*)〉 (or 〈*ξ*(*u*);(*u*, *v*)〉), because edge (*u*, *v*) is excluded from *G*_*ξ*(*u*)_ (or *G*_*ξ*(*v*)_). The tag is attached during the reduce function of UniStar in the previous round (lines 17 and 19). Given *E*_*i*_, the reduce function of UniStar first finds *m*(Λ(*u*, *G*_*i*_)) as *p*(*u*) for each node *u* ∈ *V*_*i*_ using the Rem algorithm (line 8) where *G*_*i*_ = (*V*_*i*_, *E*_*i*_) is the subgraph induced by *E*_*i*_. A node *u* ∈ *c* means edge (*u*, *p*(*u*)) has changed. For each node *v* in each connected component *p*_*u*_ of *G*_*i*_ where *u* = *m*(*p*_*u*_), UniStar emits 〈*v*, *u*〉 if *v* = *m*([*p*_*u*_]_*ξ*(*v*)_), otherwise it emits 〈*v*, *m*([*p*_*u*_]_*ξ*(*v*)_)〉 (lines 9-11, 15-19, and 22-23). If *v* = *m*([*p*_*u*_]_*ξ*(*v*)_) and *v* ∉ *c*, UniStar attaches a tag to the emitted pair 〈*v*;*u*〉 meaning that edge (*v*, *u*) is intact (lines 15-19). The tag is used for excluding intact edges in the next round.

**Algorithm 2:** UniStar-opt

**Map:** input 〈*u*;*v*〉 such that *u* > *v*

**1 if** *u* has no tag B **then**

**2**  emit 〈*ξ*(*u*);(*u*, *v*)〉

**3 if** *v* has no tag and *ξ*(*u*)≠*ξ*(*v*) **then**

**4**  emit 〈*ξ*(*v*);(*u*, *v*)〉

 **Reduce**: input 〈*i*;*E*_*i*_〉

**5** Let *G*_*i*_ = (*V*_*i*_, *E*_*i*_) be the subgraph induced by *E*_*i*_

**6** Compute |Γ^+^(*u*, *G*_*i*_)| and |Γ^⋆^(*u*, *G*_*i*_)| for each *u* ∈ *V*_*i*_ ∩ [*V*]_*i*_

**7** Clear all tags in *E*_*i*_

 /* *p*: set of key-value pairs where each key is a node *u* and the value *p*(*u*) is *m*(Λ(*u*, *G*_*i*_)) */

 /* *c*: set of keys of changed pairs in *p*: (*u*, *p*(*u*)) ∉ *E*_*i*_ if *u* ∈ *c* */

**8** *p*, *c*← findCCInPartition(*E*_*i*_)      // Rem [37] with change tracking

**9 foreach** *u*∈ the set of unique values in *p* **do**

**10**  let *p*_*u*_ be the set of all keys whose value is *u* in *p*

**11**  **foreach** *v* ∈ *p*_*u*_\{*u*} **do**

**12**   **if** *ξ*(*v*) = *i* and |Γ^⋆^(*v*, *G*_*i*_)| = |Γ^+^(*v*, *G*_*i*_)| = 0 **then**

      // case 3: do nothing

**13**   **else if** *ξ*(*u*) = *i* and |Γ^⋆^(*u*, *G*_*i*_)| = 0 **then**

**14**    emit 〈*v*, *u*〉 to ‘sep’      // case 2

**15**   **else if** *v* = *m*([*p*_*u*_]_*ξ*(*v*)_) **then**

**16**    **if** *v* ∉ *c* and *ξ*(*v*) = *i* and *ξ*(*u*) ≠ *i* **then**

**17**     emit 〈*v*;*u* with a tag〉

**18**    **else if** *v* ∉ *c* and *ξ*(*v*) ≠ *i* **then**

**19**     emit 〈*v* with a tag;*u*〉

**20**    **else if** *ξ*(*v*) = *i* and |Γ^+^(*v*, *G*_*i*_)| = 0 **the**

**21**     emit 〈*v*, *m*([*p*_*u*_]_*ξ*(*v*)_)〉 to ‘sep’      // case 1

**22**    **else**

**23**     emit 〈*v*, *m*([*p*_*u*_]_*ξ*(*v*)_)〉

### UniStar-opt: Filtering Out Dispensable Edges

In this section, we propose UniStar-opt that reduces the size of processed data significantly by filtering out *dispensable* edges. We have noticed that, even if we filter out a considerable number of edges during UniStar, UniCon is able to compute the connected components of a graph correctly. UniStar-opt excludes such filtered edges from the input of subsequent rounds so that the amount of disk and network I/Os decreases dramatically (see Fig 10). UniStar-opt filters out an edge $\left(u,v\right)\in E_{i}^{\prime }$ in three cases:

**Case 1.** *ξ*(*u*) = *ξ*(*v*) = *i* and Γ^+^(*u*, *G*_*i*_) = ∅ where Γ^+^(*u*, *G*_*i*_) = {*w*|(*w*, *u*)∈*E*_*i*_}
**Case 2.** *ξ*(*v*) = *i* and Γ^⋆^(*v*, *G*_*i*_) = ∅ where Γ^⋆^(*v*, *G*_*i*_) = {*w*|*w* has no tag, (*w*, *v*)∈*E*_*i*_}∪{*w*|*w* has no tag, (*v*, *w*)∈*E*_*i*_}
**Case 3.** *ξ*(*u*) = *i* and Γ^⋆^(*u*, *G*_*i*_) = Γ^+^(*u*, *G*_*i*_) = ∅

UniStar-opt accumulates the edges of cases 1 and 2 over several rounds into ‘sep’ and uses ‘sep’ as the input of the finishing step. The edges of case 3 are just discarded. In case 1, edge (*u*, *v*) belongs to partition *i* entirely, and node *u* has no following neighbor. By the definition of *G*_*i*_, all edges incident to node *u* in *G* are also incident to node *u* in *G*_*i*_ if *ξ*(*u*) = *i*; this fact guarantees that, if node *u* has no following neighbor in *G*_*i*_, there is no node *w* connected to *m*(Λ(*w*, *G*)) through (*u*, *v*). Thus, it is safe for UniStar-opt to exclude the edge (*u*, *v*) from the input of the next round. Note that, even if node *u* is not yet connected to *m*(Λ(*u*, *G*)), the finishing step connects *u* to *m*(Λ(*u*, *G*)) finally. Cases 2 and 3 are for the edges that remain unchanged in subsequent rounds of UniStar since they are in the connected components already discovered. In case 2, every edge incident to node *v* is intact in *G*_*i*_ where *ξ*(*v*) = *i*. In this case, *v* is *m*(Λ(*v*, *G*)) and all *w* ∈ Λ(*v*, *G*_*i*_) are directly connected to *v*. That is, the edges do not change anymore, and UniStar-opt filters them out safely. In case 3, the only edge incident to node *u* is (*u*, *v*) that is intact in *G*_*i*_ where *ξ*(*u*) = *i*. UniStar-opt discards (*u*, *v*) from $G_{i}^{\prime }$ because node *v* has no chance to connect with another node through node *u* and the same edge also exists in *G*_*ξ*(*v*)_.

**Theorem 2**. *For an edge* (*u*, *v*) *such that ξ*(*u*)≠*ξ*(*v*), *if* (*u*, *v*) *is the only edge incident to node u in G*_*ξ*(*u*)_, (*u*, *v*) *also exists in G*_*ξ*(*v*)_.

*Proof*. A logically equivalent claim: if (*u*, *v*) is in *E*_*ξ*(*u*)_, then (*u*, *v*)∈*E*_*ξ*(*v*)_ or (*u*, *w*)∈*E*_*ξ*(*u*)_ for some *w* ≠ *v*. The claim is directly true for non-intact edges by the definition of *E*_*i*_: a non-intact edge (*u*, *v*) exists in both *E*_*ξ*(*u*)_ and *E*_*ξ*(*v*)_ if *ξ*(*u*)≠*ξ*(*v*). All edges in the original graph are non-intact. Assume that a non-intact edge (*u*, *v*) changes to an intact edge in partition *ξ*(*u*) of round *r*, i.e., (*u*, *v*)∉*I*_*ξ*(*u*)_(*r* − 1) and $\left(u,v\right)\in E_{\xi (u)}\left(r\right)\cap E_{\xi (u)}^{\prime }\left(r\right)$. Edge (*u*, *v*) also exists in *E*_*ξ*(*v*)_(*r*) as (*u*, *v*) is non-intact. If (*u*, *v*) is in $E_{\xi (v)}^{\prime }\left(r\right)$, (*u*, *v*) belongs to *I*_*ξ*(*v*)_(*r*) and (*u*, *v*) exists in both *E*_*ξ*(*u*)_(*r* + 1) and *E*_*ξ*(*v*)_(*r* + 1), following the claim. If (*u*, *v*) is not in $E_{\xi (v)}^{\prime }$, it implies (*u*, *w*) is in $E_{\xi (v)}^{\prime }$ such that *w* ≠ *v*. Then, (*u*, *w*) belongs to *E*_*ξ*(*u*)_(*r* + 1), following the claim.

**Fig 5:**
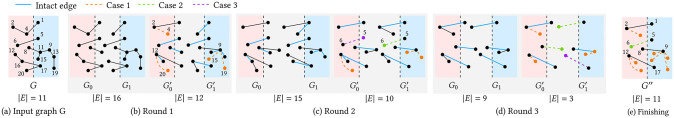
An example of UniStar-opt when the threshold *τ* = 4. Intact edges are represented as blue lines. The edges filtered by cases 1, 2, and 3 are marked with orange, green, and purple dashed lines, respectively. As the number of remaining edges after round 3 is less than *τ* = 4, the output edges of Rem on the graph induced by the remaining edges and the edges filtered by cases 1 and 2 become the input of the finishing step.

Fig 5 shows an illustration of UniStar-opt when the threshold *τ* is 4. The edges filtered by cases 1, 2, and 3 are marked with orange, green, and purple dashed lines, respectively. Blue lines are intact edges. Fig 5(a) is an input graph *G* consists of 4 connected components and 11 edges. In round 1 of Fig 5(b), edges (4, 2) and (20, 12) in $G_{0}^{\prime }$ and edges (15, 9) and (19, 9) in $G_{1}^{\prime }$ are filtered by case 1. For example, edge (4, 2) is in case 1 because node 4 has no following neighbor and nodes 2 and 4 are in partition *i*. In round 2 of Fig 5(c), edge (6, 5) in $G_{1}^{\prime }$ is filtered by case 2 because *ξ*(5) = 1 and all edges incident to node 5 in *G*_1_ is intact. In the same round, edge (6, 5) in $G_{0}^{\prime }$ is discarded by case 3 because *ξ*(6) = 0 and edge (6, 5) is intact and the only edge incident to node 6 in *G*_0_. The number of remaining edges shrinks every round quickly, and round 3 is the last round because the number of remaining edges is less than *τ* = 4. After running the Rem algorithm on the graph induced by the remaining edges, the output edges of Rem and the edges filtered by cases 1 and 2 together become the input of the finishing step as in Fig 5(e). In this example, the input and output of Rem are the same.

A MapReduce version of UniStar-opt, the optimized version of UniStar, is listed in Algorithm 2; added or modified lines from the UniStar are marked in blue. The reduce function processes the edges of the three filtering cases at lines 20-21 (case 1), lines 13-14 (case 2), and line 12 (case 3), respectively. Computing |Γ^+^(*u*, *G*_*i*_)| and |Γ^⋆^(*u*, *G*_*i*_)| for each node *u* ∈ *V*_*i*_∩[*V*]_*i*_ in advance (lines 5-6), UniStar-opt checks within a constant time that in which case each edge is.

### A hybrid map data structure

To figure out *m*(Λ(*u*, *G*_*i*_)) for each node *u*, UniStar (as well as UniStar-opt) uses the Rem algorithm with modification for tracking changes. The original Rem algorithm uses an array of size |*V*| for the mapping table *p* that maps each node *u* to *m*(Λ(*u*, *G*_*i*_)). If UniStar uses the original Rem algorithm, each worker processing a subgraph *G*_*i*_ = (*V*_*i*_, *E*_*i*_) requires |*V*| memory space for the mapping table *p* and causes an out-of-memory error when |*V*| exceeds the memory size of a worker; even though |*V*_*i*_| is much smaller than |*V*|, the array size for *p* should be |*V*| since every node has a possibility of belonging to *V*_*i*_. One easy solution to avoid an out-of-memory error is using a hash table for *p* instead of an array; it is guaranteed that the memory space required by a hash table is *O*(|*V*_*i*_|). However, accessing values by key from a hash table is 10 to 100 times slower than accessing values by index from an array.

We propose HybridMap, a data structure that guarantees fast performance and low memory usage by using the fact that *G*_*i*_ is induced by *E*_*i*_. HybridMap takes advantage of both an array and a hash table; an array is used for nodes in [*V*]_*i*_, and a hash table is used for nodes not in [*V*]_*i*_. As *E*_*i*_ is the set of edges incident to nodes in [*V*]_*i*_, most nodes in *V*_*i*_ are in [*V*]_*i*_. It suggests that most accesses to *p* are performed quickly on the array while suggesting much fewer accesses to the hash table. HybridMap requires as much memory space as the size of the array plus the number of items on the hash table. The array size equals the cardinality of [*V*]_*i*_ as the nodes are totally ordered. The number of items in the hash table is |*V*_*i*_\[*V*]_*i*_|, and it is bounded by |*E*_*i*_| = *O*(|*V*|/*ρ*); the initialization step of UniCon makes |*E*| to be *α*|*V*| for a constant *α* [34]. The hash table size |*V*_*i*_\[*V*]_*i*_| is much smaller than the array size |[*V*]_*i*_| in practice (see Section “Efficacy of HybridMap”).

**Theorem 3**. *UniStar with HybridMap requires O*((|*V*| + |*E*|)/*ρ*) *memory space to compute* $G_{i}^{\prime }$ from *G*_*i*_.

*Proof*. UniStar stores one value for each node of *V*_*i*_ in HybridMap. HybridMap uses an array for nodes in [*V*]_*i*_ and a hash table for nodes in [*V*]_*i*_\*V*_*i*_. As UniStar partitions the nodes randomly, the expected size of [*V*]_*i*_ is |*V*|/*ρ*. As *G*_*i*_ is induced by *E*_*i*_, |[*V*]_*i*_\*V*_*i*_| is at most |*E*_*i*_| when every edge contains a distinct node not belonging to [*V*]_*i*_. Assuming that the edges are evenly distributed by a random hash function, the expected size of *E*_*i*_ is 2|*E*|/*ρ*; every edge can be in two subgraphs. Thus, HybridMap requires *O*(|[*V*]_*i*_| + |[*V*]_*i*_\*V*_*i*_|) = *O*((|*V*| + |*E*|)/*ρ*).

UniStar with HybridMap avoids an out-of-memory error by setting *ρ* to be *O*((|*V*| + |*E*|)/*M*) where *M* is the memory size of a worker. Large *ρ* decreases the benefit of UniStar’s partition-aware processing and increases the running time. Thus, setting *ρ* as low as possible is good for performance, even though the running time does not increase much as *ρ* increases (see Fig 7).

## Experiments

In this section, we aim to answer the following questions from the experiments:

**Q1 Efficacy of UniStar (Section “Efficacy of UniStar”).** How much intermediate data does UniStar reduce to resolve the data explosion problem?
**Q2 Efficacy of edge filtering (Section “Efficacy of Edge Filtering”).** How many edges are filtered by the edge filtering of UniStar-opt?
**Q3 Efficacy of HybridMap (Section “Efficacy of HybridMap”).** How efficient is UniCon using HybridMap compared to using an array or a hash table?
**Q4 Scalability (Section “Scalability”).** How does UniCon scale up in terms of the number of machines and the data size?
**Q5 Performance on real-world datasets (Section “Results on Real-world Datasets”).** How well does UniCon perform on real-world graphs compared to state-of-the-art algorithms?

### Experimental settings

#### Datasets

We evaluate UniCon with real-world graphs summarized in Table 2. TW is a follower-followee network in Twitter. LJ and FS are friendship networks in social networking services LiveJournal and Friendster, respectively. SD is a domain level hyperlink network. GSH, CW, and HL are page level hyperlink networks. RMAT-*k* for *k* ∈ {21, 23, 25, 27, 29, 31, 33} is a synthetic graph following RMAT model [43], and we generate it using TeGViz [44], a distributed graph generator. We set RMAT parameters (a, b, c, d) to (0.57, 0.19, 0.19, 0.05).

**Table 2:** The summary of datasets.

Dataset	# nodes	# edges	Source
LJ	4,847,571	68,993,773	SNAP
TW	41,652,230	1,468,365,182	Kwak et al. [45]
FS	65,608,366	1,806,067,135	SNAP
SD	89,247,739	2,043,203,933	Web Data Commons
GSH	988,490,691	33,877,399,152	WebGraph
CW	6,257,706,595	71,746,553,402	LemurProject
HL	3,443,082,324	128,736,914,167	Web Data Commons
RMAT-21	1,114,816	31,457,280	7* N/A (Synthetic graphs)
RMAT-23	4,120,785	125,829,120	
RMAT-25	15,212,447	503,316,480	
RMAT-27	56,102,002	2,013,265,920	
RMAT-29	207,010,037	8,053,063,680	
RMAT-31	762,829,446	32,212,254,720	
RMAT-33	1,090,562,291	128,849,018,880	

#### Machines

The cluster used in the experiment consists of 10 machines, and each machine is equipped with Intel Xeon E3-1220 CPU (4-cores at 3.10GHz), 16GB RAM, and 2 SSDs of 1TB. Hadoop v3.2.1, Spark v3.0.1, and MPICH v3.3 are installed. One machine of the cluster acts as the master and also as a worker, and the others act as workers. Single machine algorithms are tested on the master node.

#### Algorithms

We implement three versions of UniCon (UniCon-naïve, UniCon-base, UniCon-opt) on Hadoop. For a fair comparison, we use the original codes from the authors of the competitors: Cracker, FastSV, LACC, PowerGraph, PACC, and ConnectIt. All codes are publicly available on the Web. Rem is implemented with C++11.

- UniCon-naïve: the naïve version of the proposed method described in Section “UniStar-naïve”.
- UniCon-base: the proposed method in Section “UniStar”.
- UniCon-opt: the proposed method in Section “UniStar-opt: Filtering Out Dispensable Edges”.
- PACC [34]: the state-of-the-art MapReduce algorithm.
- Cracker [30]: a MapReduce algorithm implemented in Apache Spark. We add Cracker here because Cracker and PACC have not been tested together so far.
- PowerGraph [22]: the connected component algorithm implemented on PowerGraph, a representative pregel-like distributed-memory graph processing system.
- LACC [24], FastSV [25]: the state-of-the-art distributed-memory algorithms.
- ConnectIt [15]: the state-of-the-art multi-core algorithm. UF-Rem-CAS and LDD sampling are used since the combination is the fastest according to [15].
- Rem [37]: the fastest sequential algorithm based on Union-Find according to [35].

#### Parameters

**Fig 6:**
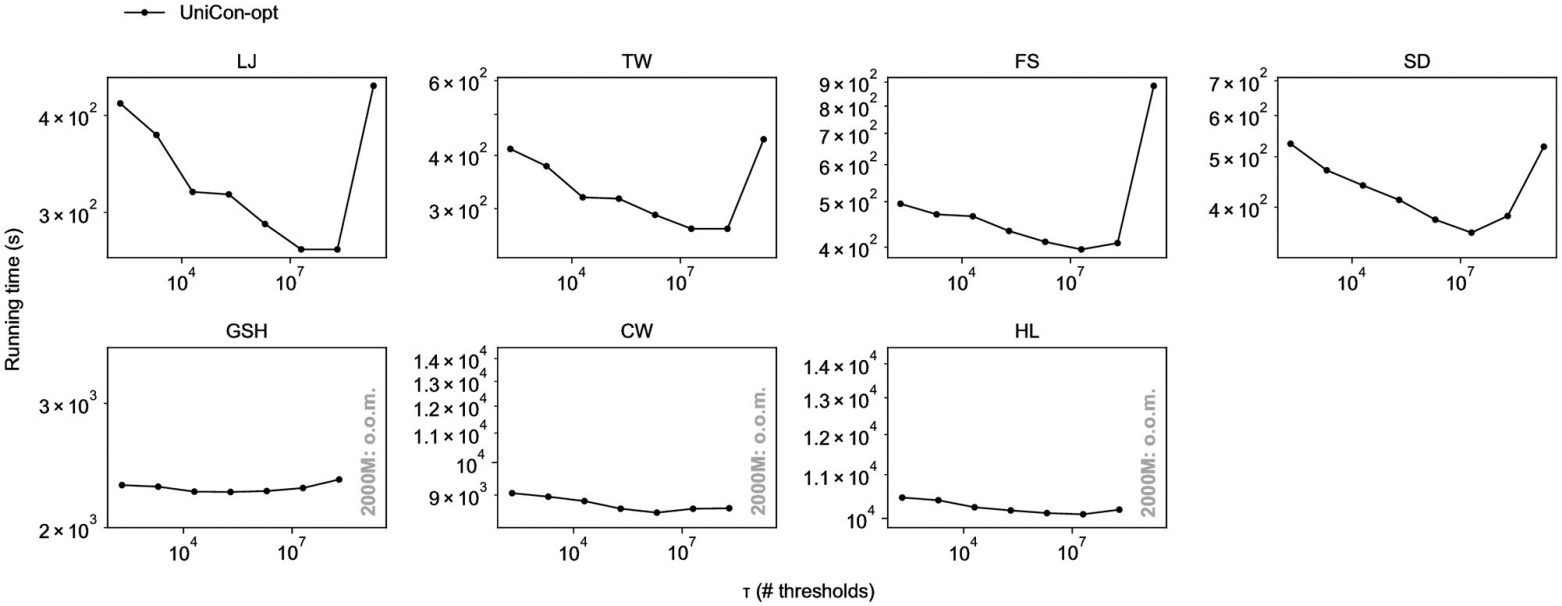
The running time of UniCon-opt on various *τ*.

Unless otherwise noted, we use all 10 workers. To find the optimal condition for each method, we vary the parameter values and compare the running time. Fig 6 shows the effect of threshold *τ* on the running time of UniCon-opt on each dataset. When processing GSH,CW, and HL with *τ* = 2000*M*, UniCon-opt gets an out-of-memory error as it tries to handle large data using only a single machine. We use the optimal *τ* for UniCon-opt unless otherwise noted.

**Fig 7:**
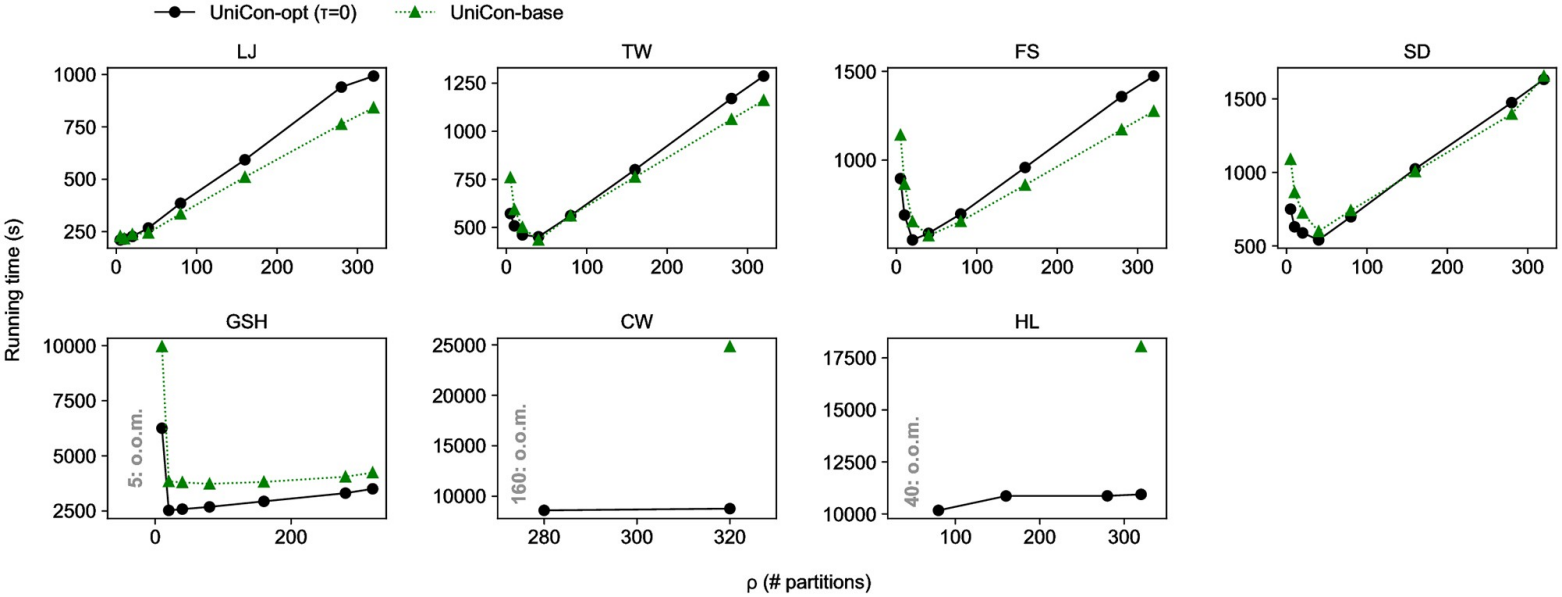
The running time of UniCon-opt and UniCon-base on various partition number *ρ*. With large data like GSH, CW, and HL, when *ρ* is greater than each optimal value, the running time of UniCon increases marginally as *ρ* increases.

Fig 7 shows the running time of UniCon-opt and UniCon-base with various partition numbers *ρ*. The running time soars when *ρ* is too small because the methods do not exploit all the workers. Also, the running time tends to increase as *ρ* increases because of computational overhead, but the increase in running time is marginal when the graph is large enough e.g., GSH, CW, and HL. It implies that, when the graph is enormous, UniCon can avoid an out-of-memory error by increasing *ρ*, with a slight increase in running time. Accordingly, we set the number of partition *ρ* to 280 for CW. Both PACC and Cracker perform the best when the number of partitions is 20, and PowerGraph, LACC, and FastSV do the best when the number of processors is 10, 4, and 36, respectively; thus, we use them as the default values.

### Efficacy of UniStar

**Fig 8:**
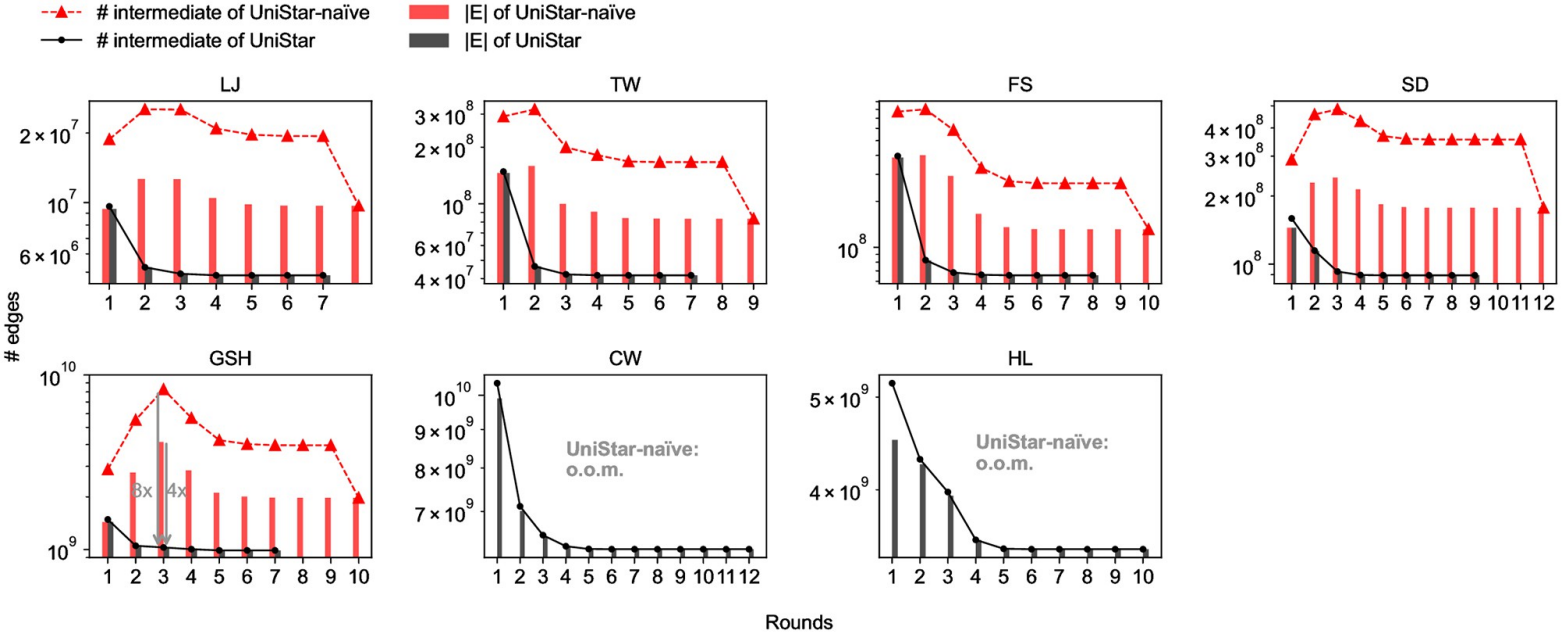
The input and intermediate data sizes of UniStar and UniStar-naïve each round. UniStar reduces both sizes by up to 4× and 8×, respectively, compared to UniStar-naïve.

We compare UniCon-base and UniCon-naïve to show the effects made by UniStar; UniCon-base uses UniStar and UniCon-naïve does UniStar-naïve. For the two operations, Fig 8 shows the input and intermediate data sizes each round where the intermediate data is the output of the Map step in the MapReduce implementation. Thanks to the partition-aware processing and excluding intact-edges, UniStar effectively resolves the data explosion problem that UniStar-naïve suffers from. UniStar reduces the input data and intermediate data sizes significantly up to 4× and 8×, respectively. UniStar-naïve fails to process CW and HL because of massive intermediate data.

**Fig 9:**
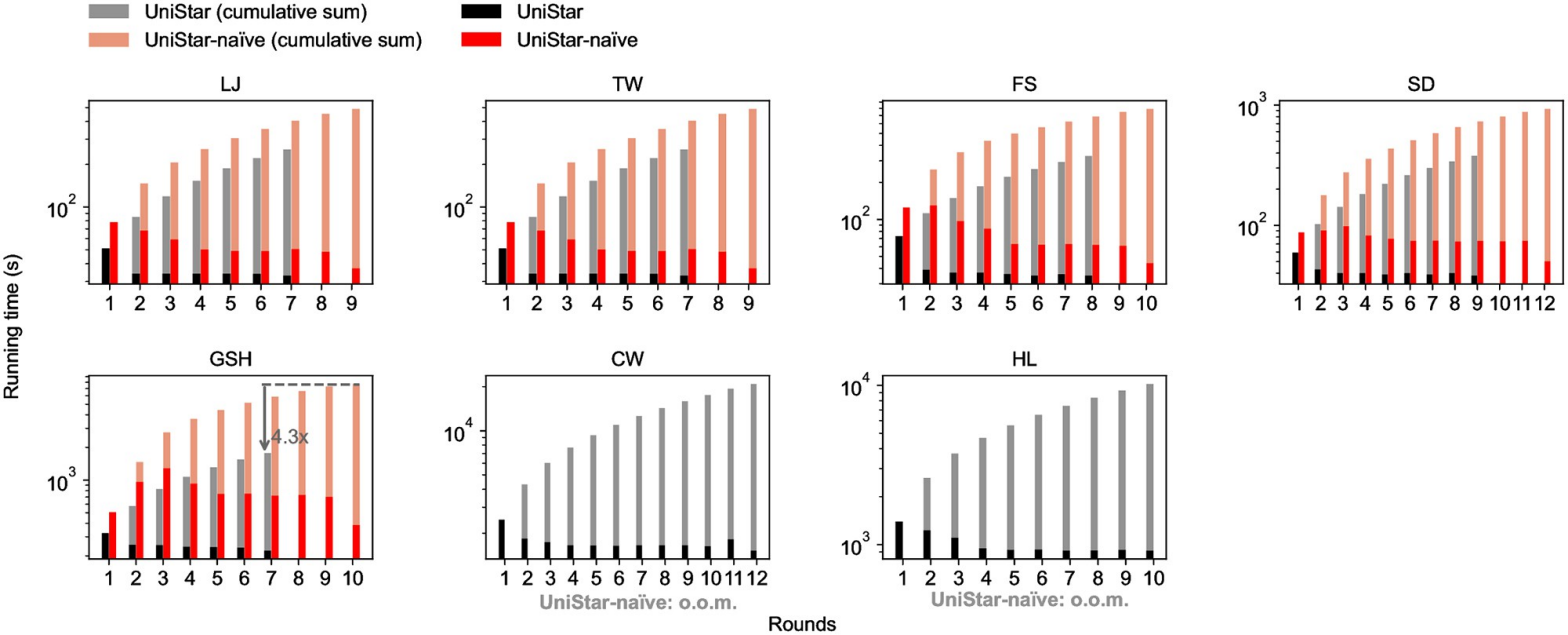
The running time of UniStar and UniStar-naïve, and its’ cumulative sums, respectively. UniStar is faster than UniStar-naïve in all rounds and requires fewer rounds.

Fig 9 shows the running time of UniStar and UniStar-naïve, and the cumulative sums of them. UniStar is 2-5 times faster than UniStar-naïve in every case and UniCon-base takes fewer rounds than UniCon-naïve; accordingly, UniCon-base reduces the total running time by up to 4.3× compared to UniCon-naïve.

### Efficacy of edge filtering

**Fig 10:**
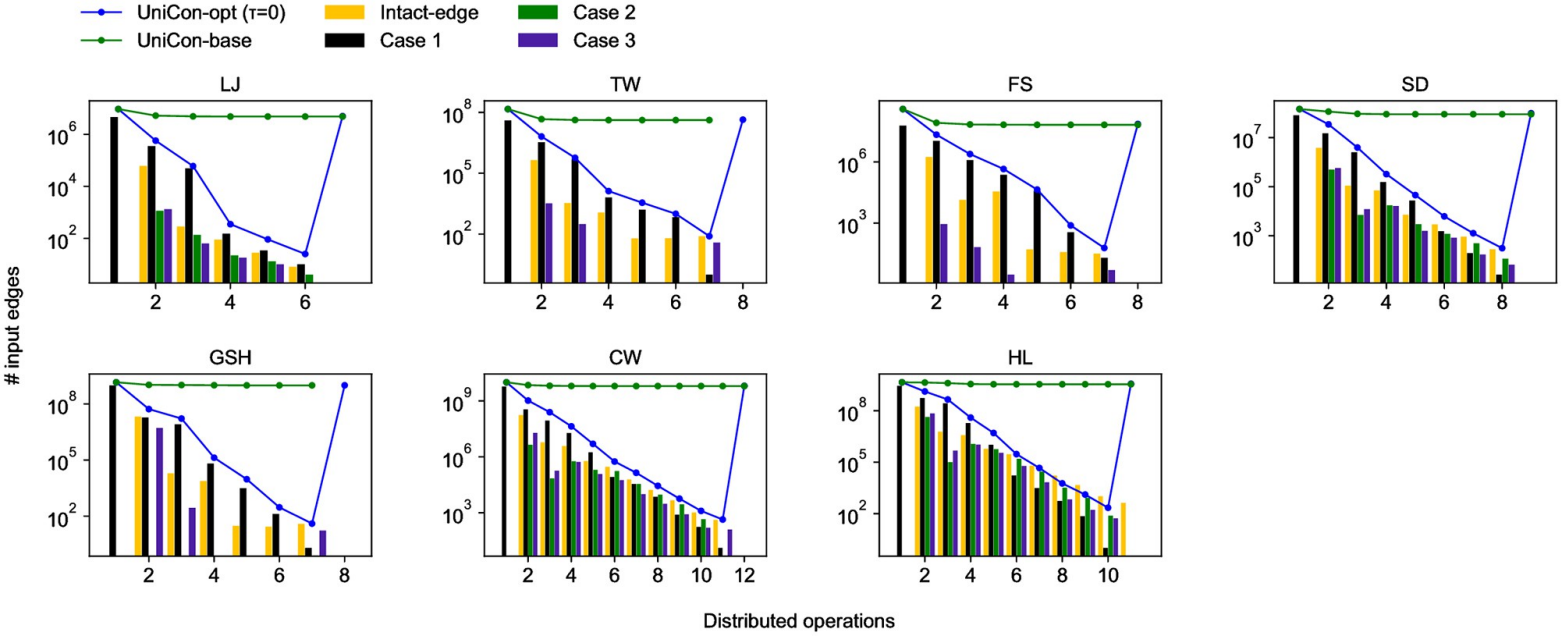
The number of input edges (denoted by lines) for UniCon-opt (*τ* = 0) and UniCon-base each round. Filtering dispensable edges (denoted by bars), UniCon-opt shrinks the input size by 80.4% on average every round on CW.

The lines in Fig 10 show the numbers of input edges to UniCon-opt (*τ* = 0) and UniCon-base each round. We fix *τ* to be 0 to show the efficacy of edge filtering, which is applied to UniCon-opt. The edge filtering of UniCon-opt decreases the input size rapidly for every dataset. Meanwhile, UniCon-base, which is not accompanied by edge filtering, takes a huge amount of input every round. In round 12 of UniCon-opt, the number of input edges increases because the last round of UniCon-opt is the finishing step; it takes as input the output edges of the partitioning step and filtered edges by cases 1 and 2. UniCon-opt shrinks the input size by 80.4% on average every round. The bars in four colors represent the decreased input sizes by four cases: excluded intact edges (Section “UniStar: The Unified Star Operation”) and filtered edges by three cases in Section “UniStar-opt: Filtering Out Dispensable Edges”. All four cases contribute greatly to reducing the size of the input data. Note that TW, FS, and GSH have only one connected component, so there are no edges filtered by case 2, in which edges belonging to early discovered connected components are filtered.

**Fig 11:**
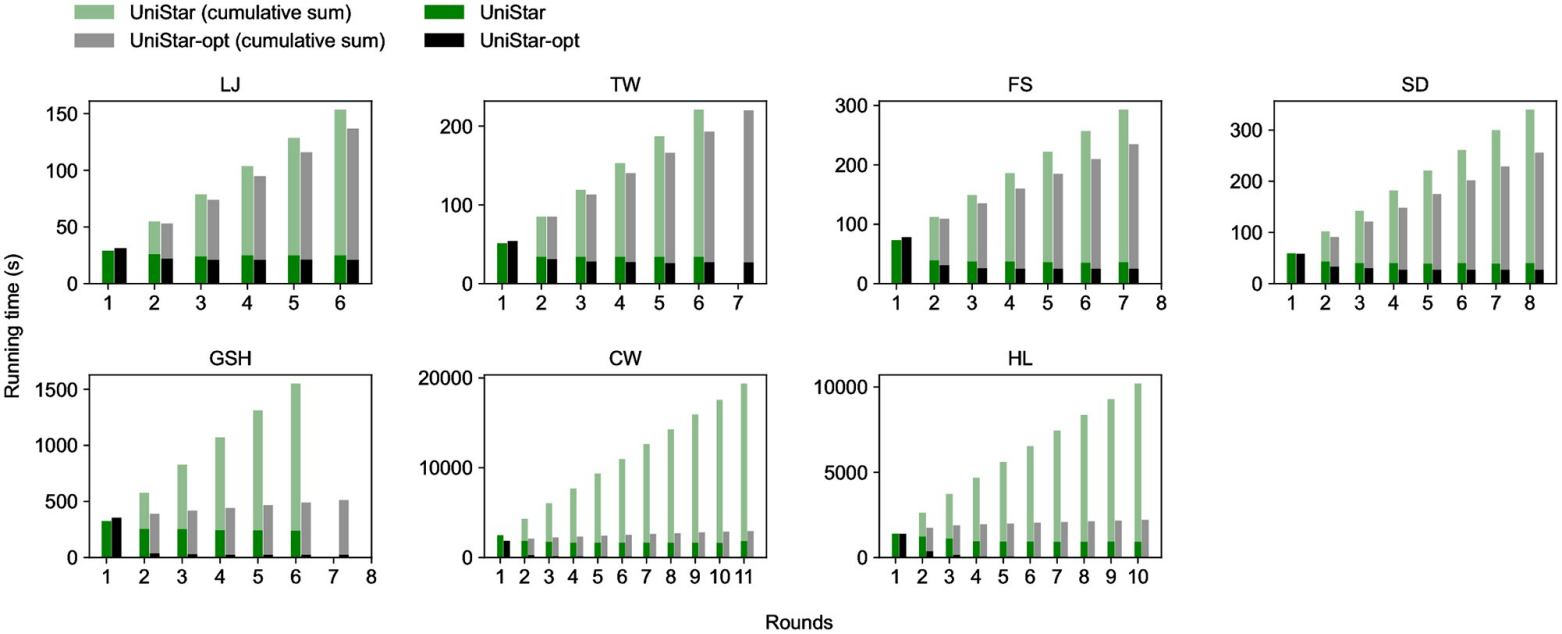
The running time of UniStar-opt and UniStar each round. By the edge filtering, the running time of UniStar-opt drops quickly.

Fig 11 shows the running time of UniStar-opt (*τ* = 0) and UniCon-base, and the cumulative sums of them each round. The running time of UniStar-opt drops dramatically as the input size plummets every round.

### Efficacy of HybridMap

**Fig 12:**
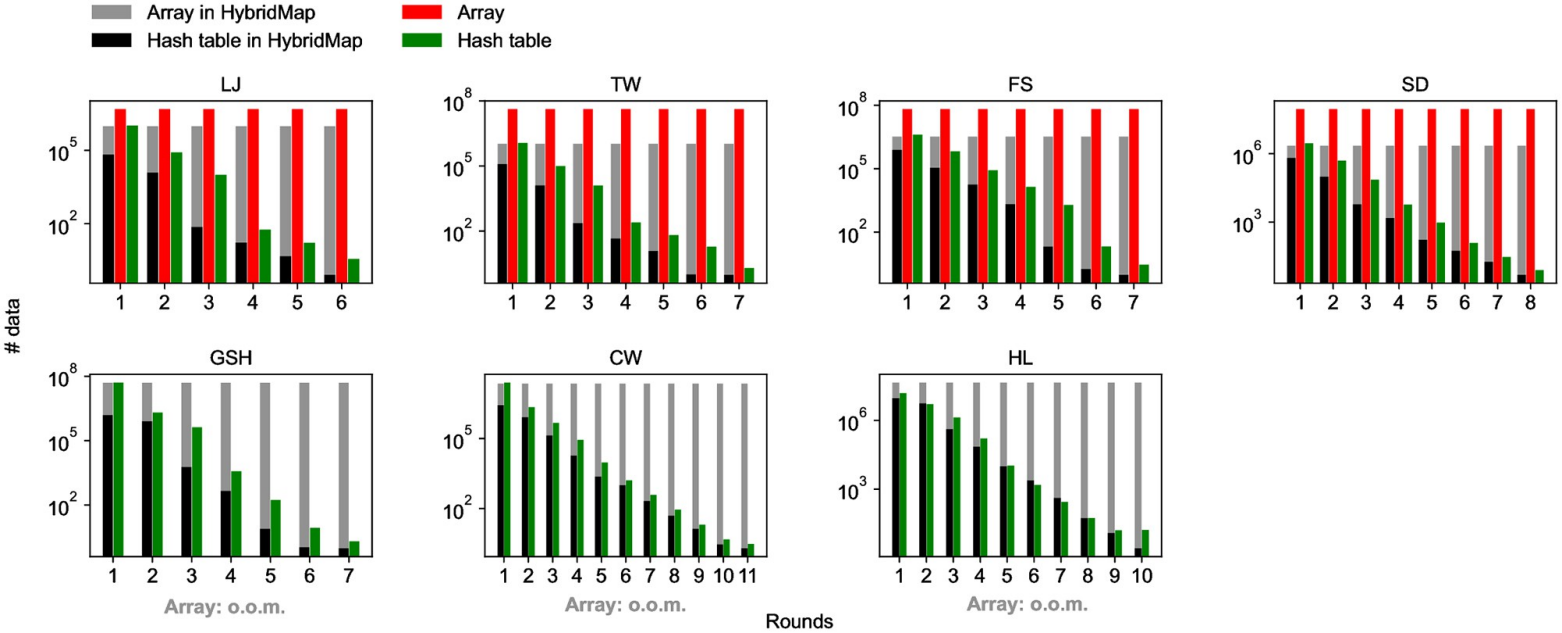
The numbers of data stored in HybridMap, a hash table, and an array each round of UniCon-opt. The Y-axis is in a log scale. HybridMap significantly reduces the data stored in memory compared to an array, letting UniCon-opt succeed in processing large graphs: GSH, CW, and HL. HybridMap and a hash table have no significant difference in terms of the peak number of stored data.

**Fig 13:**
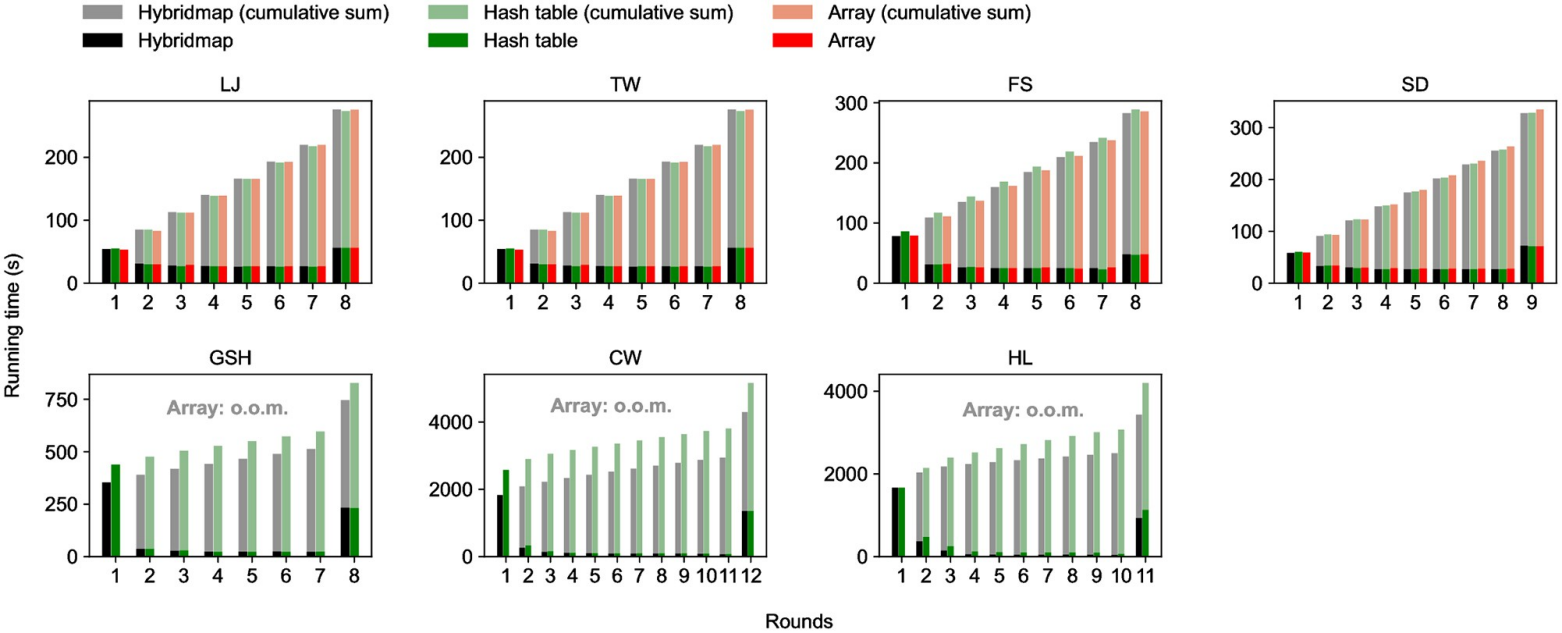
The running time of UniStar-opt with HybridMap, a hash table, and an array, respectively, each round. UniCon-opt with HybridMap outperforms Unicon-opt with hash tables when the graph is large enough (FS, SD, GSH, CW, and HL) thanks to fast random accesses of HybridMap. UniCon-opt with arrays fails on large graphs (GSH, CW, and HL) because of an out-of-memory error.

UniCon-opt uses HybridMap to keep the connectivity of nodes in each partition. To show the effectiveness of HybridMap, we compare the original UniCon-opt with the versions where HybridMap is replaced with arrays and hash tables, respectively. Fig 12 shows the average size of data that UniCon-opt (*τ* = 0) stores in memory on each worker, and Fig 13 does the running time of UniCon-opt when the underlying data structure used by UniCon-opt is HybridMap, an array, and a hash table, respectively. HybridMap only takes *O*((|*V*| + |*E*|)/*ρ*) for each worker each round as analyzed theoretically in Theorem 3, while UniCon-opt with arrays fails in processing GSH, CW, and HL because |*V*| of GSH, CW, and HL exceeds the memory size of a worker. Although HybridMap takes more space than a stand-alone hash table, it is sufficiently small already, and HybridMap reduces the running time by using an array for frequently accessed nodes. The size of data actually stored in the hash table of HybridMap is much smaller than the theoretical result *O*(|*E*|/*ρ*). UniCon-opt with HybridMap shows the best performance in terms of speed every round, and its cumulative sum is also the lowest, except on LJ and TW. UniCon-opt with arrays shows the long running time even though an access to array is much faster than an access to a hash table because it loads an array of size |*V*| to every worker, taking a long time for memory allocation. The running time of UniCon-opt with HybridMap is 22.7% is lower than UniCon-opt with hash tables on CW.

### Scalability

**Fig 14:**
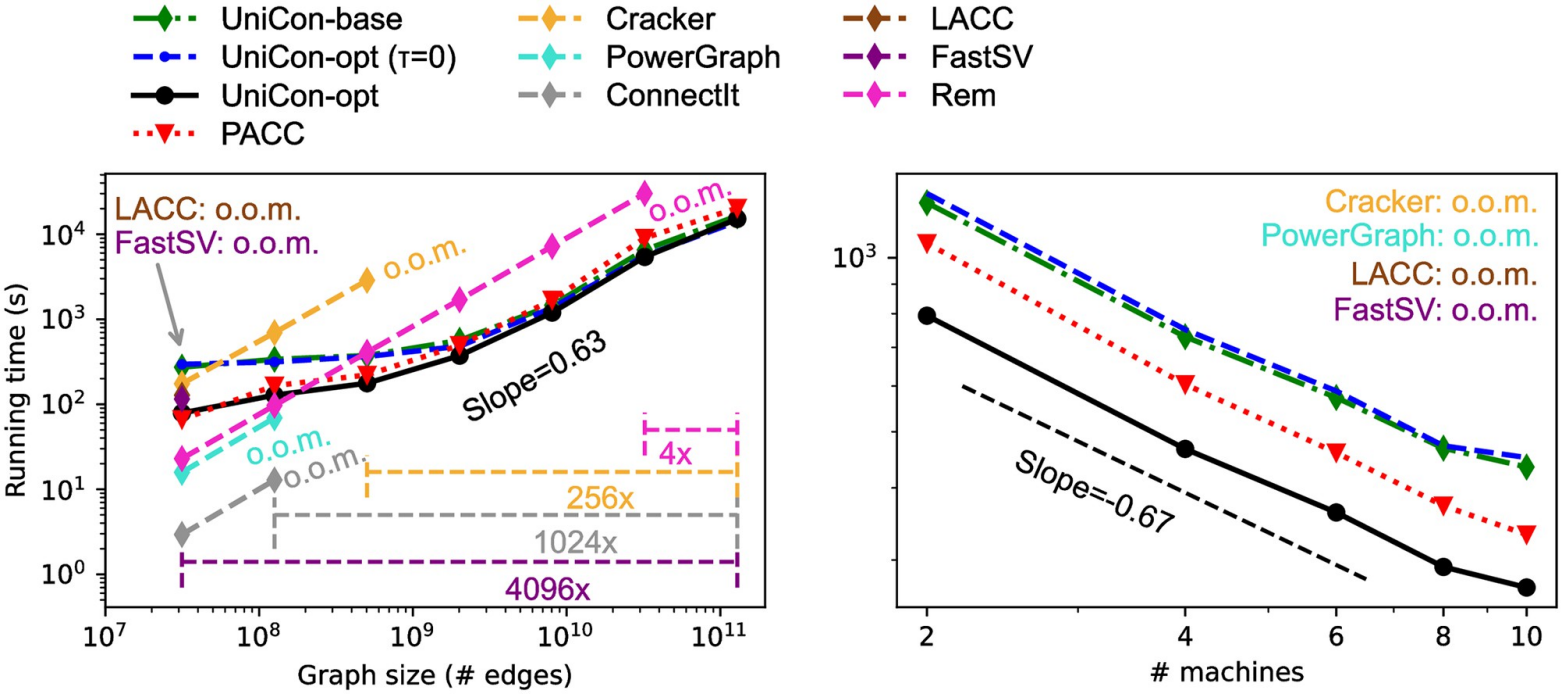
Data and machine scalability. (left) UniCon handles up to 4096× larger graphs than competitors. (right) UniCon-opt shows the best performance regardless of the number of machines.

Fig 14 shows (left) the data scalability and (right) the machine scalability of all algorithms in Section “Algorithms”. In the left figure, RMAT graphs with various sizes are used for the data scalability analysis. Both axes are in a log scale. UniCon and PACC show sub-linear scalability with slopes under 1. This can be interpreted as Hadoop takes a long time when processing small data due to the start-up and clean-up overheads. Distributed-memory algorithms (PowerGraph, LACC, FastSV), Cracker, ConnectIt, and Rem show near-linear scalability but fail on medium-sized graphs because of out-of-memory errors. As a result, UniCon processes 4096× larger graph than LACC and FastSV, 1024× larger graph than PowerGraph and ConnectIt, 256× larger graph than Cracker, and 4× larger graph than Rem. Even though Rem is a sequential algorithm, Rem handles larger graphs than distributed-memory algorithms because Rem stores only nodes in memory while the distributed-memory algorithms require to store the entire graph in memory. According to papers [25] and [15], LACC, FastSV, and ConnectIt reportedly can process graphs with 100 billion edges when expensive machines are available (e.g., a supercomputer with 262K cores for LACC and FastSV, and an expensive server computer with 72 cores and 1TB memory for ConnectIt). On a commodity cluster of 10 cheap machines used in this experiment, however, they cannot even process a graph containing only a billion edges.

In the right figure, the machine scalability analysis shows the running time on a various number of machines. TW is used. Both axes are in a log scale. All distributed-memory algorithms and MapReduce algorithms are tested, but Cracker, PowerGraph, LACC, and FastSV are omitted here because they fail to process TW because of out-of-memory errors. UniCon-opt shows the best performance regardless of the number of machines. The slope of UniCon-opt from 2 to 10 machines is -0.67, meaning that the running time decreases by 1.59× when the number of machines doubles.

### Results on Real-world Datasets

**Fig 15:**
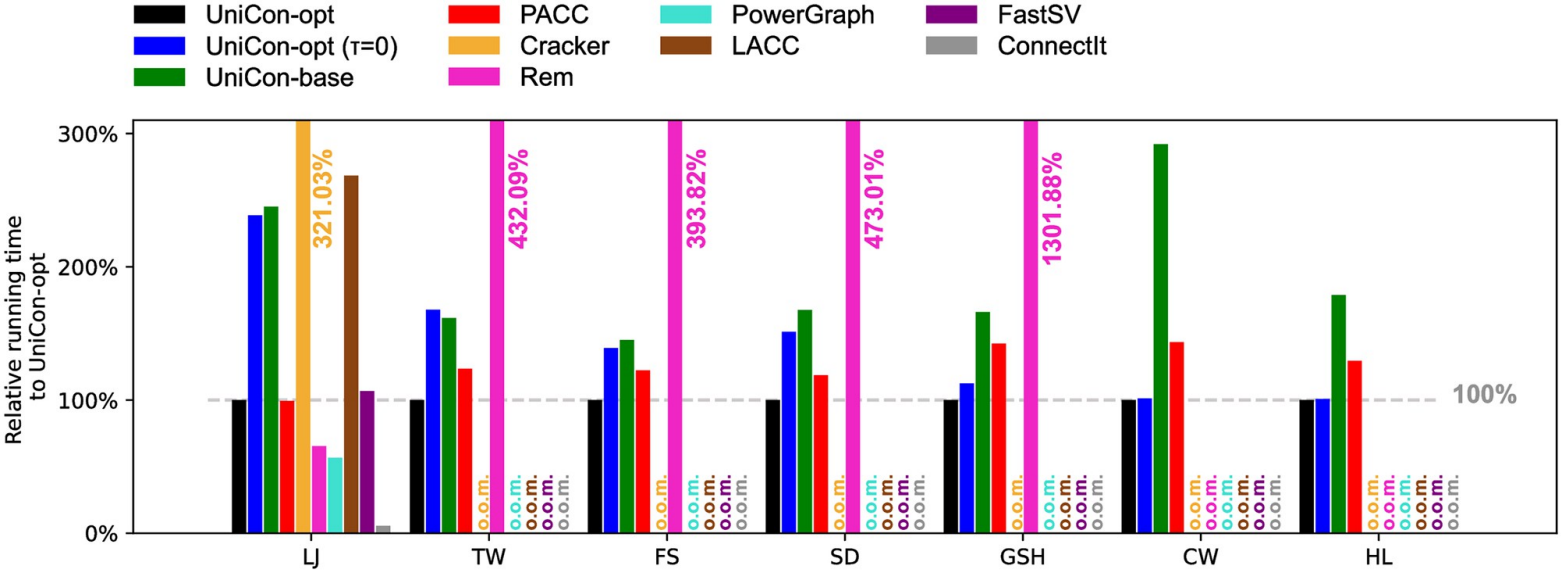
The relative running time, compared to UniCon-opt, of competitors on real-world graphs. o.o.m.: out-of-memory error. UniCon-opt with optimal *τ* outperforms all competitors on all graphs except for LJ. Rem, ConnectIt, and PowerGraph are faster than UniCon-opt on LJ but fail on all other graphs because of out-of-memory errors.

Fig 15 shows the relative running time, compared to UniCon-opt, of all the algorithms in Section “Algorithms” on the real-world graphs listed in Table 2. UniCon-opt shows the best performance on all graphs except LJ. All distributed-memory algorithms (PowerGraph, LACC, FastSV), ConnectIt, and Cracker fail on all graphs except LJ because of out-of-memory errors. Even on LJ, UniCon-opt is faster than LACC and FastSV, while ConnectIt is the fastest. Only UniCon and PACC succeed in processing CW, the largest real-world graph tested in this experiment. UniCon-opt outperforms PACC for all graphs; the speed of UniCon-opt is 143% of PACC’s.

**Fig 16:**
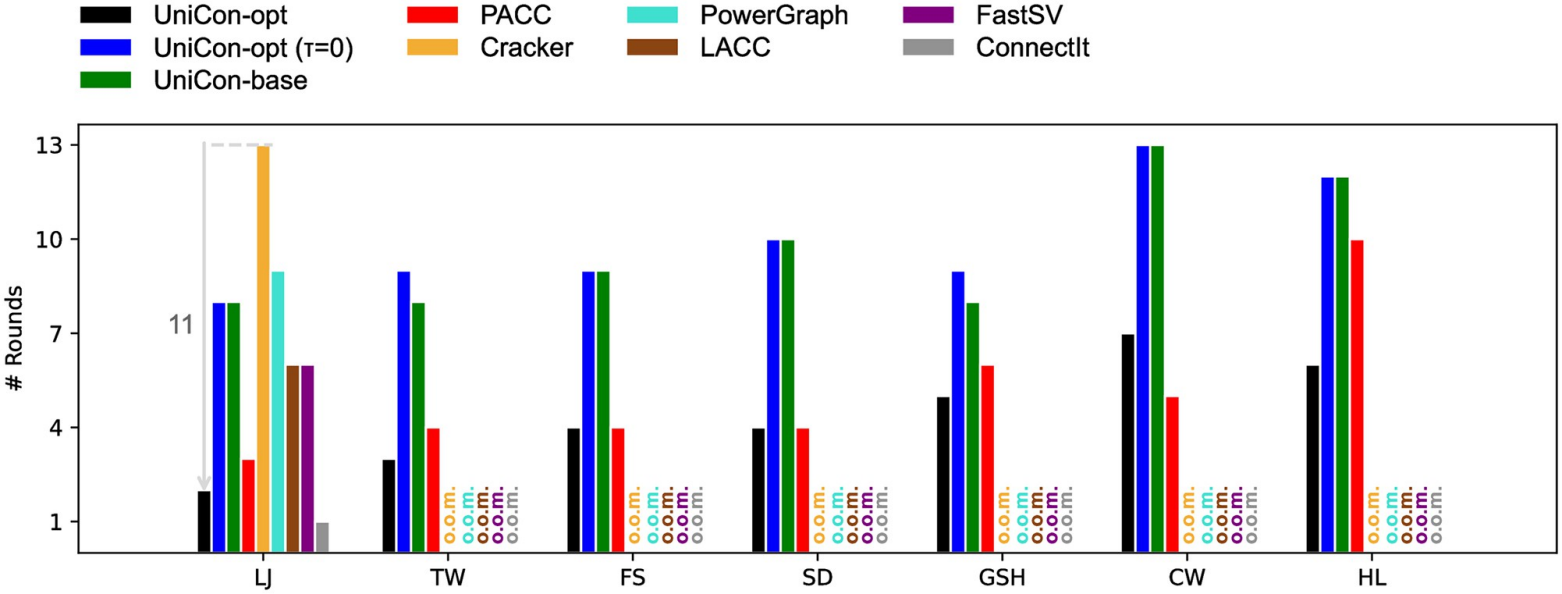
The numbers of rounds required by all algorithms except Rem on real-world graphs. UniCon-opt requires up to 11 fewer rounds than competitors.

Fig 16 shows the numbers of rounds required by all algorithms except Rem on real-world graphs. For PACC, one execution of a star-operation is counted as one round. Thanks to partition-aware processing, UniCon-opt requires a smaller number of rounds than PACC, Cracker, FastSV, and LACC, and reduces up to 11 rounds compared to competitors. UniCon-opt runs more rounds than PACC on CW because the optimal *τ* = 2*M* of Unicon-opt is small. It implies that UniStar-opt is more efficient than PACC’s star-operations, and thus UniCon-opt performs a single machine algorithm only when the data size is reduced sufficiently.

## Conclusion

In this paper, we propose UniStar, a unified star-operation, and UniCon, a new distributed algorithm finding connected components in an enormous graph using UniStar. The partition-aware processing of UniStar effectively avoids the data explosion problem reducing the intermediate data size by up to 87.5% compared to UniStar-naïve. Edge filtering of UniCon shrinks the size of input data by 80.4% on average each round. The HybridMap data structure of UniCon ensures that the memory consumption of each worker is *O*((|*V*| + |*E*|)/*ρ*) where *ρ* is the number of partitions and improves performance by 22.7% over when using a typical hash table. As a result, on a commodity cluster, UniCon handles up to 4096 times larger graphs than graphs competitors can process. With a cluster of only 10 cheap machines, UniCon succeeds in processing a graph containing 129 billion edges, showing the fastest performance.
